# Supplementary material for: Iterative improvement in the automatic modular design of robot swarms
Source: PeerJ Comput Sci. 2020 Dec 7;6:e322. doi: 10.7717/peerj-cs.322 (PMC7924708; doi:10.7717/peerj-cs.322)
Supplement: Supplemental Information 3 [file peerj-cs-06-322-s003.zip › argos3/doc/api/standalone/a00399_source.html]

ARGoS: core/utility/plugins/factory.h Source File


- Main Page
- Related Pages
- Namespaces
- Classes
- Files

- File List
- File Members

# core/utility/plugins/factory.h

Go to the documentation of this file.

```
00001 
00044 #ifndef FACTORY_H
00045 #define FACTORY_H
00046 
00047 #include <argos3/core/utility/configuration/argos_exception.h>
00048 #include <map>
00049 #include <iostream>
00050 #include <string>
00051 #include <cstdlib>
00052 
00053 namespace argos {
00054 
00058    template<typename TYPE>
00059    class CFactory {
00060 
00061    public:
00065       typedef TYPE* TCreator();
00066 
00070       struct STypeInfo {
00071          TCreator* Creator;
00072          std::string Author;
00073          std::string Version;
00074          std::string BriefDescription;
00075          std::string LongDescription;
00076          std::string Status;
00077       };
00078 
00082       typedef std::map<std::string, STypeInfo*> TTypeMap;
00083 
00084    public:
00089       static TTypeMap& GetTypeMap();
00090 
00101       static void Register(const std::string& str_label,
00102                            const std::string& str_author,
00103                            const std::string& str_version,
00104                            const std::string& str_brief_desc,
00105                            const std::string& str_long_desc,
00106                            const std::string& str_status,
00107                            TCreator* pc_creator);
00113       static TYPE* New(const std::string& str_label);
00114 
00119       static bool Exists(const std::string& str_label);
00120 
00125       static void Print(std::ostream& c_os);
00126 
00130       static void Destroy();
00131    };
00132 
00133 /*
00134  * Include the actual template implementation
00135  */
00136 #include <argos3/core/utility/plugins/factory_impl.h>
00137 
00149 #define REGISTER_SYMBOL(BASECLASS,                                      \
00150                         CLASSNAME,                                      \
00151                         LABEL,                                          \
00152                         AUTHOR,                                         \
00153                         VERSION,                                        \
00154                         BRIEF_DESCRIPTION,                              \
00155                         LONG_DESCRIPTION,                               \
00156                         STATUS)                                         \
00157    namespace argos {                                                    \
00158       extern "C" {                                                      \
00159          BASECLASS* BASECLASS ## CLASSNAME ## Creator() {               \
00160             return new CLASSNAME;                                       \
00161          }                                                              \
00162       }                                                                 \
00163       class C ## BASECLASS ## CLASSNAME ## Proxy {                      \
00164       public:                                                           \
00165       C ## BASECLASS ## CLASSNAME ## Proxy() {                          \
00166          CFactory<BASECLASS>::                                          \
00167             Register(LABEL,                                             \
00168                      AUTHOR,                                            \
00169                      VERSION,                                           \
00170                      BRIEF_DESCRIPTION,                                 \
00171                      LONG_DESCRIPTION,                                  \
00172                      STATUS,                                            \
00173                      BASECLASS ## CLASSNAME ## Creator);                \
00174       }                                                                 \
00175       };                                                                \
00176       C ## BASECLASS ## CLASSNAME ## Proxy BASECLASS ## CLASSNAME ## _p; \
00177    }
00178 
00179 }
00180 
00181 #endif
```

---

Generated on 10 Jul 2018 for ARGoS by 
 1.6.1
